# Combined Ethanol Extract of Grape Pomace and Omija Fruit Ameliorates Adipogenesis, Hepatic Steatosis, and Inflammation in Diet-Induced Obese Mice

**DOI:** 10.1155/2013/212139

**Published:** 2013-04-18

**Authors:** Su-Jung Cho, Un Ju Jung, Hae-Jin Park, Hye-Jin Kim, Yong Bok Park, Sang Ryong Kim, Myung-Sook Choi

**Affiliations:** ^1^Department of Food Science and Nutrition, Kyungpook National University, 1370 Sankyuk Dong, Puk-ku, Daegu 702-701, Republic of Korea; ^2^Center for Food and Nutritional Genomics Research, Kyungpook National University, 1370 Sankyuk Dong, Puk-ku, Daegu 702-701, Republic of Korea; ^3^Food R&D, CJ Cheiljedang Corp., Seoul 152-051, Republic of Korea; ^4^School of Life Sciences & Biotechnology, Kyungpook National University, Daegu 702-701, Republic of Korea

## Abstract

The aim of this study was to evaluate the long-term effects of grape pomace ethanol extract (GPE) with or without omija fruit ethanol extract (OFE) on adiposity, hepatic steatosis, and inflammation in diet-induced obese mice. Male C57BL/6J mice were fed a high-fat diet (HFD) as the control diet and HFD plus GPE (0.5%, w/w) with or without OFE (0.05%, w/w) as the experimental diet for 12 weeks. GPE alone did not significantly affect adipogenesis and hepatic steatosis. However, the supplementation of GPE + OFE significantly lowered body weight gain, white adipose tissue weight, adipocyte size, and plasma free fatty acid and adipokines (leptin, PAI-1, IL-6, and MCP-1) levels in HFD-fed mice compared to those of the control group. These beneficial effects of GPE + OFE were partly related to the decreased expression of lipogenic and inflammatory genes in white adipose tissue. GPE + OFE supplementation also significantly lowered liver weight and ameliorated fatty liver by inhibiting expression of hepatic genes involved in fatty acid and cholesterol syntheses as well as inflammation and by activating hepatic fatty acid oxidation. These findings suggest that the combined ethanol extract of grape pomace and omija fruit has the potential to improve adiposity and fatty liver in diet-induced obese mice.

## 1. Introduction

Obesity, a metabolic disease characterized by an excessive accumulation of fat in white adipose tissue (WAT), is associated with chronic inflammation and is considered a risk factor of nonalcoholic fatty liver disease (NAFLD). WAT secretes various inflammation-related adipocytokines, and the dysregulated production of adipocytokines promotes NAFLD. In particular, visceral WAT is directly linked to the severity of hepatic inflammation and fibrosis in NAFLD, independent of hepatic steatosis [1]. Furthermore, visceral WAT might promote NAFLD by the release of free fatty acids that are delivered directly into the portal vein [2]. There is a close relationship between visceral and liver fat contents in obese and nonobese individuals [3–5]. Lipogenesis is also an important metabolic pathway regulating adipose and hepatic fat accumulation [6].

It is well known that phytochemicals-rich foods like grapes and their products prevent metabolic diseases such as obesity, NAFLD, and insulin resistance. Grape pomace, a byproduct of wine processing, consists mainly of peels, stems, and seeds and accounts for about 20% of the weight of the grapes processed into wine [7]. The antioxidant and anti-inflammatory effects of grape pomace ethanol extract (GPE) in mice fed a high-fat diet (HFD) were reported [8]. In HFD-fed rats, GPE also ameliorated obesity-induced insulin resistance by inhibiting hepatic lipogenic, gluconeogenic, and inflammatory gene expression [9]. However, little is known about whether GPE can protect against lipid accumulation and inflammation in the WAT and liver of diet-induced obese mice. Therefore, we examined the effects of GPE on the expression profiles of genes related to lipogenesis and inflammation in the WAT and liver, and plasma adipocytokine levels in HFD-fed C57BL/6J mice, which are a good model for diet-induced obesity that display many of the characteristics of the human disease including visceral obesity, hyperglycemia, and hyperinsulinemia [10]. 

We also hypothesized that combined supplementation of GPE and omija fruit extract (OFE) may synergistically ameliorate visceral fat accumulation, hepatic steatosis, and inflammation in diet-induced obese mice. Fructus Schisandrae, the fruit of omija (*Schisandra chinensis* Baillon), is another phytochemical food which has antioxidant, anti-inflammatory, and antimetabolic properties. Omija has been traditionally used as a tonic, sedative, and antidiabetic agent in Asia and is a main compound of traditional herbal medicine which consists of several herbs [11, 12]. Taeyeumjoweetang, a traditional Korea herbal medicine consisting of eight herbs including omija, suppressed body weight and serum leptin and resistin levels in mice [13] and improved serum and hepatic lipid profiles in HFD-fed mice [14]. In addition, a recent study reported that dibenzocyclooctadiene lignans isolated from *Schisandra chinensis* had a fatty acid synthase inhibitory effect [15]. 

Accordingly, we investigated the effect of GPE alone or combined with OFE on adiposity, hepatic steatosis, and inflammation in HFD-induced obese mice and the underlying mechanisms based on lipid metabolism and inflammation in the WAT and liver.

## 2. Materials and Methods

### 2.1. Preparation of Extracts

Grapes (*Vitis vinifera*, MBA (Muscat Bailey A) species) and omija (*Schisandra chinensis* Baillon) were purchased from Yeongcheon-si (Gyeongsangbuk-do, Korea) and Mungyeong-si (Gyeongsangbuk-do, Korea), respectively. In this study, grape pomace (skin and stem) and omija fruits (Fructus Schisandrae) were used. Samples were prepared by adding 2 L of 80% and 50% ethanol to 100 g of dried grape pomace and omija fruit, respectively; extraction was done at 80°C for 2 h and then cooled. The solution was filtered (Whatman paper no. 2), concentrated with a rotary vacuum evaporator, and stored at −70°C. The final weight of the lyophilized powder of grape pomace ethanol extract (GPE) was 33.9 g (33.9%) and omija fruit ethanol extract (OFE) was 39.7 g (39.7%). Resveratrol is a representative compound in grape pomace and schizandrin is a typical compound in omija. The GPE included 0.2 mg/g resveratrol, 52 mg/g flavonoids, and 6 mg/g catechins (3.3 mg/g catechin, 2.6 mg/g epicatechin). The OFE contained 8 mg/g schizandrin and 7 mg/g flavonoids. The content of flavonoid, resveratrol, and schizandrin of grape pomace and omija extracts was 48 mg/g, 0.2 mg/g, and 55 mg/g, respectively. 

### 2.2. Animal and Diets

Male mice (strain C57BL/6J) were purchased from the Jackson Laboratory (Bar Harbor, ME, USA) at 4 weeks of age. The animals were individually housed with a constant temperature (24°C) and 12 h light/dark cycle and fed a pelletized commercial nonpurified diet for 1 week after arrival. The mice were then randomly divided into 3 groups (*n* = 10) and fed the control and experimental diets for 12 weeks: high-fat diet control (HFD, 20% high-fat diet based on AIN-76 diet plus 1% cholesterol, w/w), grape pomace extract (GPE, HFD combined with 0.5% grape pomace extract, w/w), and the combined extracts of grape pomace and omija fruit (GPE + OFE, HFD combined with 0.5% grape pomace extract, and 0.05% omija fruit extract, w/w). The composition of the diets is presented in Table 1. The mice had free access to food and distilled water during the experimental period. Their food intake and body weight were measured daily and weekly, respectively. 

At the 12th week, mice were anaesthetized with diethyl ether and sacrificed after 12 h of fasting. Blood was taken from the inferior vena cava and then centrifuged at 1000 ×g for 15 min at 4°C, and the plasma was separated to analyze plasma biomarkers. After blood collection, the liver and adipose tissues were promptly removed, rinsed, weighed, frozen in liquid nitrogen, and stored at −70°C. This animal study protocol was approved by the Ethics Committee for animal studies at Kyungpook National University, Republic of Korea.

### 2.3. Plasma Adipocytokine and Aminotransferase Levels

To measure the plasma adiponectin and adipsin levels, we used a quantitative sandwich enzyme immunoassay kit (ELISA kit, Millipore, MA, USA). The levels of plasma cytokines (interleukin (IL)-6, monocyte chemotactic protein-1 (MCP-1), tumor necrosis factor (TNF)-*α*), leptin, resistin, and plasminogen activator inhibitor-1 (PAI-1) were determined with a multiplex detection kit from Bio-Rad (Hercules, CA). All samples were assayed in duplicate and analyzed with a Luminex 200 Labmap system (Luminex, Austin, TX). Data analyses were done with Bio-Plex Manager software version 4.1.1 (Bio-Rad, USA). The plasma alanine aminotransferase (ALT) and aspartate aminotransferase (AST) were determined using enzymatic kits (Asan, Seoul, Republic of Korea).

### 2.4. Plasma and Hepatic Lipid Contents

Plasma lipid concentrations were determined with commercially available kits: total cholesterol, triglyceride, HDL-cholesterol (Asan, Seoul, Republic of Korea), free fatty acids, and phospholipids (Wako Chemicals, Richmond, VA, USA). The hepatic lipids were extracted using the method of Folch et al. [16] and hepatic lipid levels were analyzed with the same enzymatic kits used in the plasma analyses.

### 2.5. RNA Extraction and Real-Time Quantitative PCR Analysis

Total RNA was isolated from the liver and WAT using TRIzol reagent (Invitrogen Life Technologies, Grand Island, NY) according to the manufacturer's instructions. DNase digestion was used to remove any DNA contamination, and RNA was reprecipitated in ethanol to ensure no phenol contamination. The RNA purity and integrity were evaluated with the Agilent 2100 Bioanalyzer (Agilent Technologies, Palo Alto, USA). Equal amounts of RNA from each experimental group were pooled to normalize individual differences. Total RNA (1 *μ*g) was reverse-transcribed into cDNA using the QuantiTect reverse transcription kit (Qiagen, Germany). Then mRNA expression was quantified by real-time quantitative PCR, using the QuantiTects SYBR green PCR kit (Qiagen, Germany) on the CFX96TM real-time PCR system (Bio-Rad, UK). The sequences of the primers were as follows: ACAT (acetyl coenzyme A acetyltransferase), 5′-AGAAATCAAGCAAAGATCCA-3′ (forward), 5′-AGGAGTCCTTGGGTAGTTGT-3′ (reverse); ACC1 (acetyl CoA carboxylase 1), 5′-GGACAGACTGATCGCAGAGAAAG-3′ (forward), 5′-TGGAGAGCCCCACACACA-3′ (reverse); ACOX1 (acyl CoA oxidase 1), 5′-CCCAACTGTGACTTCCATT-3′ (forward), 5′-GGCATGTAACCCGTAGCACT-3′ (reverse); CD36, 5′-TGGTGGATGGTTTCCTAGCCTTTC-3′ (forward), 5′-TCGCCAACTCCCAGGTACAATC-3′ (reverse); CPT1 (carnitine palmitoyl transferase 1), 5′-ATCTGGATGGCTATGGTCAAGGTC-3′ (forward), 5′-GTGCTGTCATGCGTTGGAAGTC-3′ (reverse); FAS (fatty acid synthase), 5′-CGCTCCTCGCTTGTCGTCTG-3′ (forward), 5′-AGCCTTCCATCTCCTGTCATCATC-3′ (reverse); GAPDH (glyceraldehyde-3-phosphate dehydrogenase), 5′-ACAATGAATACGGCTACAGCAACAG-3′ (forward), 5′-GGTGGTCCAGGGTTTCTTACTCC-3′ (reverse); HMGR (3-hydroxy-3-methyl-glutaryl-CoA reductase), 5′-TTCACGCTCATAGTCGCTGGATAG-3′ (forward), 5′-TGGTTCAATTCTCTTGGACAATCTTC-3′ (reverse); IL-6, 5′-GAGGATACCACTCCCAACAGACC-3′ (forward), 5′-AAGTGCATCATCGTTGTTCATACA-3′ (reverse); LPL (lipoprotein lipase), 5′-CTGACCAAGGATAGTGGGATATAG-3′ (forward), 5′-GGTAACTGAGCGAGACTGTGTCT-3′ (reverse); MCP-1, 5′-TTCCTCCACCACCATGCAG-3′ (forward), 5′-CCAGCCGGCAACTGTGA-3′ (reverse); ME (malic enzyme), 5′-AGGGCACATTGCTTCAGTTC-3′ (forward), 5′-TGTACAGGGCCAGTTTACCC-3′ (reverse); NF-*κ*B (nuclear factor kappa B), 5′-GAAGTGAGAGAGTGAGCGAGAGAG-3′ (forward), 5′-CGGGTGGCGAAACCTCCTC-3′ (reverse); PGC1*α* (peroxisome proliferator-activated receptor *γ* co-activator 1 *α*), 5′-AAGTGTGGAACTCTCTGGAACTG-3′ (forward), 5′-GGGTTATCTTGGTTGGCTTTATG-3′ (reverse); PGC1*β* (peroxisome proliferator-activated receptor *γ* co-activator 1 *β*), 5′-GGTCCCTGGCTGACATTCAC-3′ (forward), 5′-GGCACATCGAGGGCAGAG-3′ (reverse); PPAR*α* (peroxisome proliferator-activated receptor *α*), 5′-CCTGAACATCGAGTGTCGAATAT-3′ (forward), 5′-GGTCTTCTTCTGAATCTTGCAGCT-3′ (reverse); PPAR*γ* (peroxisome proliferators-activated receptor *γ*), 5′-ACCACTCGCATTCCTTTGAC-3′ (forward), 5′-CCACAGACTCGGCACTCAAT-3′ (reverse); SCD1 (stearoyl-CoA desaturase 1), 5′-CCCCTGCGGATCTTCCTTAT-3′ (forward), 5′-AGGGTCGGCGTGTGTTTCT-3′ (reverse); TNF-*α*, 5′-GCAGGTCTACTTTAGAGTCATTGC-3′ (forward), 5′-TCCCTTTGCAGAACTCAGGAATGG-3′ (reverse); and UCP2 (uncoupling protein 2), 5′-ACCAAGGGCTCAGAGCATGCA-3′ (forward), 5′-TGGCTTTCAGGAGAGTATCTTTG-3′ (reverse). Cycle thresholds were determined based on the SYBR green emission intensity during the exponential phase. The fold changes were calculated using the 2-ΔΔCt method; transcripts of GAPDH were also amplified from the samples in order to validate the internal control genes for real-time quantitative PCR detection.

### 2.6. Hepatic Fatty Acid Oxidation

Enzyme sources were prepared according to the method developed by Hulcher and Oleson [17] with slight modification. Fatty acid *β*-oxidation (*β*-oxidation) was measured spectrophotometrically by monitoring the reduction of NAD to NADH in the presence of palmitoyl CoA as described by Lazarow [18], with slight modification. The amount of protein in the enzyme sources was determined with the Bradford method using bovine serum albumin as the standard.

### 2.7. Histopathological Analysis

The liver and epididymal WAT were fixed in 10% formalin buffer solution and then routinely processed for paraffin embedding. The 4 *μ*m sections of each tissue were stained with hematoxylin eosin (H&E) and the stained tissues were observed under an optical microscope (Zeiss Axioscope, Germany) with a magnifying power of ×200. The epididymal adipocyte size was measured by using Motic Images Plus 2.0 ML (Motic). 

### 2.8. Statistical Analysis

The statistical analyses were performed with the statistical package for social science software program (SPSS Inc., Chicago, IL, USA). Significant differences between the means were determined by one-way ANOVA. Duncan's multiple-range test was performed if differences were identified between the groups at *P* < 0.05. All data are expressed as the means with their standard error of the mean.

## 3. Results

### 3.1. Food Intake, Body Weight Gain, and WAT Weight and Size

**Figure 1:**
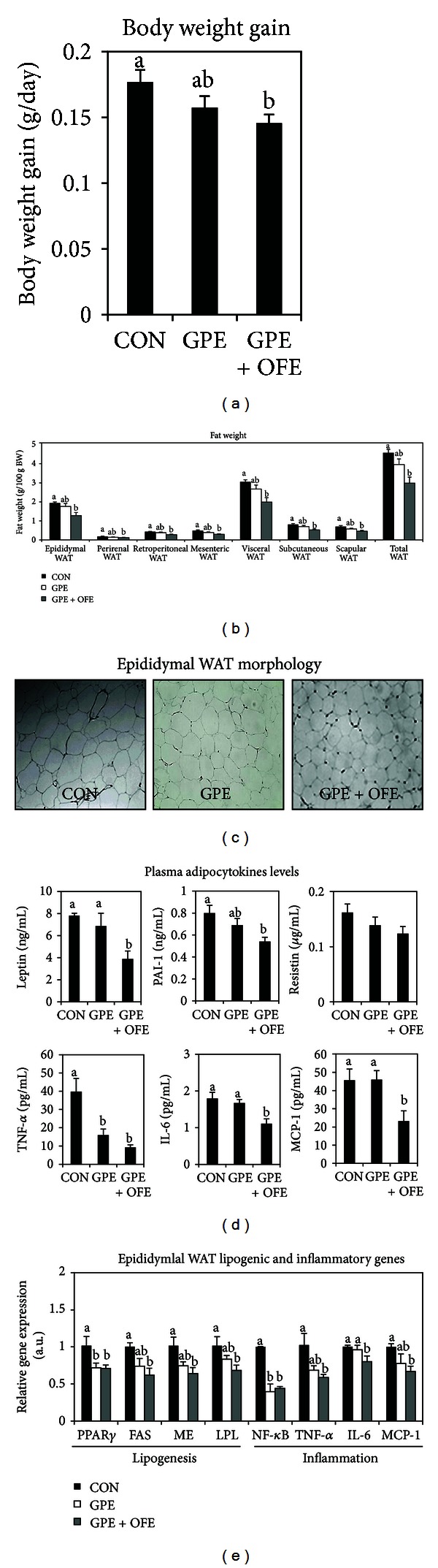
Effect of GPE alone or combined with OFE on body weight gain (a), body fat weight (b), epididymal WAT morphology (c), plasma adipocytokine levels (d) and epididymal WAT lipogenic and inflammatory gene expressions (e) in HFD-fed mice. (a), (b), (d), and (e) Data are the Means ± SE (*n* = 10).   ^ab^Means not sharing a common letter are significantly different among the groups at *P* < 0.05. (c) Representative photomicrographs of epididymal WAT are shown at ×200 magnification (*n* = 10). CON: high-fat diet control; GPE: high fat diet plus grape pomace extract (0.5%, w/w); GPE + OFE: high fat diet plus grape pomace extract (0.5%, w/w) combined with omija fruit extract (0.05%, w/w); WAT: white adipose tissue; PPAR*γ*: peroxisome proliferator-activated receptor *γ*; FAS: fatty acid synthase; ME: malic enzyme; LPL: lipoprotein lipase; NF-*κ*B: nuclear factor-*κ*B; TNF-*α*: tumor necrosis factor-*α*; IL-6: interleukin-6; MCP-1: monocyte chemotactic protein-1.

Supplementation with GPE did not significantly reduce body weight gain, WAT weight, and WAT cell size in mice fed a HFD (Figures 1(a)–1(c)). However, mice supplemented with GPE + OFE showed a significantly lower body weight gain compared to the HFD control (Figure 1(a)). Furthermore, supplementation with GPE + OFE markedly reduced the weight of visceral WAT (including epididymal, perirenal, retroperitoneal, and mesenteric WAT), subcutaneous WAT, scapular WAT, and total WAT as well as epididymal adipocyte size in HFD-fed mice (Figures 1(b) and 1(c)). There was no significant change in food intake among the groups (Table 1).

### 3.2. Plasma Adipokine Levels

Similar to the results for body weight gain and body fat, supplementation with GPE + OFE significantly lowered plasma leptin and PAI-1 levels compared to the HFD control group (Figure 1(d)). Furthermore, the combination of GPE and OFE significantly lowered plasma TNF-*α*, IL-6, and MCP-1 levels in the HFD-fed mice. The plasma TNF-*α* level was also lowered in the GPE group compared to the HFD control group. Mice supplemented with GPE + OFE exhibited a 24% lower plasma resistin level compared to the HFD control mice, although the differences were not statistically significant. Plasma adiponectin and adipsin levels were not significantly altered by GPE alone or with OFE (Table 1). 

### 3.3. Expression of Genes Involved in Adipogenesis and Inflammation in Epididymal WAT

We investigated the potential mechanisms by which the combination of GPE and OFE might attenuate the HFD-induced activation of adipogenesis in the epididymal WAT of mice (Figure 1(e)). Supplementation with GPE alone or with OFE significantly downregulated the mRNA level of adipogenic transcription factor, PPAR*γ*, in the epididymal WAT of HFD-fed mice. Additionally, the mRNA levels of several key adipogenic target genes, FAS, ME, and LPL, were significantly downregulated in the GPE + OFE-supplemented mice compared to the HFD control mice. Mice supplemented with GPE also showed lower mRNA levels of adipogenic genes, FAS (26%), ME (27%), and LPL (18%), compared to the HFD control mice, but the differences were not statistically significant. 

Next, the mRNA expression of key genes involved in inflammation was measured in the epididymal WAT of mice (Figure 1(e)). Supplementation with GPE resulted in a significantly lower NF-*κ*B mRNA level in epididymal WAT, and it tended to lower the epididymal TNF-*α* and MCP-1 mRNA levels by 33% and 23%, respectively, compared to the HFD control group. GPE + OFE also significantly downregulated the mRNA levels of proinflammatory cytokine genes, such as TNF-*α*, IL-6, and MCP-1, as well as transcription factor, NF-*κ*B, in epididymal WAT compared to the HFD control mice. 

### 3.4. Plasma and Hepatic Lipid Levels

There were no significant differences in plasma triglyceride, phospholipid, and total-cholesterol levels among the groups (Table 2). However, mice supplemented with GPE showed a significantly higher level of plasma HDL cholesterol and a lower atherogenic index (AI) compared to the HFD control mice. Supplementation with GPE + OFE also tended to increase the plasma HDL-cholesterol level and significantly lower the AI value compared to the control group. Furthermore, the plasma free fatty acid level was significantly lowered in the GPE + OFE group only, but not in the GPE group, compared to the HFD control group. 

**Figure 2:**
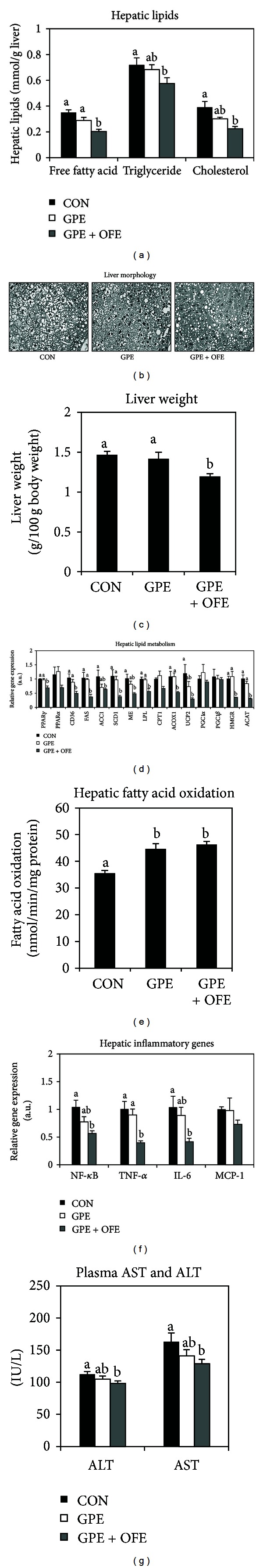
Effect of GPE alone or combined with OFE on hepatic lipid contents (a), liver morphology (b), liver weight (c) and hepatic lipogenic and inflammatory gene expressions and fatty acid oxidation (d–f) in HFD-fed mice. (a), (c)–(f) Data are the Means ± SE (*n* = 10). ^ab^Means not sharing a common letter are significantly different among the groups at *P* < 0.05. (b) Representative photomicrographs of the liver are shown at ×200 magnification (*n* = 10). CON: high-fat diet control; GPE: high fat diet plus grape pomace extract (0.5%, w/w); GPE + OFE: high fat diet plus grape pomace extract (0.5%, w/w) combined with omija fruit extract (0.05%, w/w); WAT: white adipose tissue; PPAR*γ*, peroxisome proliferator-activated receptor *γ*; FAS: fatty acid synthase; ME: malic enzyme; LPL: lipoprotein lipase; HMGR: 3-hydroxy-3-methyl-glutaryl-CoA reductase; ACAT: acyl-CoA cholesterol acyl transferase; NF-*κ*B: nuclear factor-*κ*B; TNF-*α*: tumor necrosis factor-*α*; IL-6: interleukin-6; MCP-1: monocyte chemotactic protein-1.

The contents of hepatic free fatty acid, triglyceride, and cholesterol were significantly lower in the GPE + OFE group than in the control group, whereas GPE alone had no significant effect on these hepatic lipid contents (Figure 2(a)). Morphological analysis of the liver also indicated that the combination of GPE and OFE supplementation markedly decreased lipid accumulation shown by the decreases in both the number and size of hepatic lipid droplets compared to the HFD control group, and the liver weight in GPE + OFE was lower than that in the control group (Figures 2(b) and 2(c)). Overall, dietary GPE + OFE supplementation can ameliorate HFD-mediated hepatic steatosis in diet-induced obese mice. 

### 3.5. Hepatic Lipogenic and Inflammatory Gene Expression, Fatty Acid Oxidation, and Plasma AST and ALT

We determined the mRNA expression of genes involved in lipogenesis as well as fatty acid oxidation in the liver to investigate how GPE + OFE ameliorates hepatic fat accumulation (Figures 2(d) and 2(e)). The mRNA expressions of genes for fatty acid uptake and *de novo* fatty acid synthesis, including CD36, FAS, ACC1, SCD1, ME, and LPL, were markedly lower in the GPE + OFE-supplemented mice than in the HFD control mice. Furthermore, mRNA expressions of key cholesterol regulating genes (HMGR and ACAT) as well as fatty acid oxidation-related genes (ACOX1 and UCP2) were significantly lowered in the GPE + OFE group than in the control mice. In contrast, hepatic fatty acid *β*-oxidation was significantly elevated in both the GPE and GPE + OFE groups than that of the control group. 

Next, we explored the effect of GPE alone or with OFE on hepatic inflammatory gene expression (Figure 2(f)). The mRNA expression of the proinflammatory cytokines, such as IL-6 and TNF-*α*, was significantly downregulated in the liver of the GPE + OFE mice compared to the control mice. GPE + OFE-supplemented mice also showed a significant decrease in hepatic NF-*κ*B mRNA expression, but GPE alone did not significantly alter the expression of these proinflammatory genes in the liver. 

We also investigated whether the GPE + OFE can protect the liver from HFD-induced liver damage (Figure 2(g)). Supplementation with GPE + OFE group significantly reduced plasma ALT and AST levels compared to the HFD control mice. Mice supplemented with GPE also exhibited 7% and 14% lower plasma ALT and AST levels compared to the HFD control mice although differences were not statistically significant. These results indicate that HFD-associated liver dysfunction could be ameliorated by GPE + OFE.

## 4. Discussion

The present study first examined whether the long-term supplementation of GPE alone or with OFE could induce protective effects against HFD-induced obesity and NAFLD in C57BL/6J mice. To investigate the underlying mechanisms, we focused on the expression profiles of genes related to lipogenesis and inflammation in the WAT and liver, and on the levels of plasma adipocytokines. In the present study, we demonstrated that the combination of GPE and OFE led to a favorable effect on adiposity, hepatic steatosis, and inflammation in HFD-induced obese mice, although GPE alone did not significantly decrease body weight, body fat, and hepatic lipid accumulation.

A previous study reported that GPE supplementation (approximately 450 mg/kg body weight/day) did not significantly suppress HFD-induced body weight gain in rats [9]. Recently, Hogan et al. [8] also demonstrated that supplementation with GPE (approximately 250 mg/kg body weight/day) in a HFD for 12 weeks did not affect body weight gain but reduced the levels of proinflammatory maker and C-reactive protein in the plasma of mice. In agreement with these previous studies, we observed that dietary GPE (approximately 470 mg/kg body weight/day) did not induce a significant lowering effect on body weight gain and adiposity, but the plasma TNF-*α* level and epididymal NF-*κ*B mRNA expression were significantly lower in the GPE group than those in the control group, suggesting dietary GPE has a potential antiinflammatory effect.

In contrast to GPE alone, the combination of GPE and OFE significantly lowered body weight gain, WAT weight and WAT adipocyte size in mice fed a HFD. The decrease in WAT weight, and adipocyte size observed in the GPE + OFE-supplemented obese mice was associated with the downregulated mRNA expression of genes involved in* de novo* fatty acid syntheses, FAS and ME, in epididymal WAT. FAS is a key enzyme responsible for *de novo *biosyntheses of long-chain fatty acids from acetyl CoA and malonyl CoA in the presence of NADPH, and ME generates NADPH to be consumed in fatty acid syntheses. Expression of both enzymes is regulated at the transcriptional level and is sensitive to nutritional and hormonal regulation, [19, 20]. In addition, the decrease in mRNA expression of LPL and PPAR*γ* could also contribute to the amelioration of adiposity with supplementation of GPE and OFE in HFD-fed mice. LPL is expressed at high levels in adipose tissue and hydrolyzes circulating triglyceride-rich lipoproteins (very low-density lipoproteins and chylomicrons) to generate free fatty acids, which are subsequently reesterified for storage as triglycerides in adipocytes [21]. The level of LPL mRNA was significantly increased in the WAT of HFD-fed mice and obese subjects [22, 23], whereas WAT-specific LPL deficiency diminished body weight and fat mass in *ob/ob* mice although it increased endogenous fatty acid syntheses in WAT [24]. PPAR*γ* is a main transcription factor that regulates many adipocyte genes encoding proteins and enzymes involved in adipogenesis and lipid metabolism, including fatty acid syntheses, fatty acid uptake and storage [25]. Collectively, the decreased mRNA expression of adipogenic genes (FAS, ME, and LPL) and their transcription factor (PPAR*γ*) in response to GPE plus OFE seemed to contribute to a significant reduction in body fat accumulation in the HFD-induced obese mice. Similarly, a traditional Korean herbal medicine that includes omija (Taeumjowitang) significantly lowered body weights, body fat, and serum leptin level in HFD-fed mice [13] and obese Korean children [26], and another pilot study that is evaluating the effect of Taeumjowitang on obesity in Korean adults is currently in progress [27]. We also found that a 12-week dietary supplementation with Taeumjowitang at two doses (1.5%, 3%, w/w) dose-dependently lowered body  weight and body fat mass in mice fed a HFD (60% kcal from fat based on AIN-93G diet) (Choi J. Y. and Choi M. S., unpublished data 2013). Furthermore, one preliminary study in exercise-trained rats fed a AIN-76 semisynthetic diet containing two doses (0.002 and 0.006 g/kg body weight) of OFE for 6 weeks demonstrated beneficial effects on body fat accumulation in a dose-dependent manner (Kim Y. J., Jung U. J., and Choi M. S., unpublished data 2010-2011). The body fat lowering effect of OFE was related to the decreased activity of adipose enzymes involved in fatty acid syntheses (fatty acid synthase and malic enzyme). Notably, the dose of OFE tested in the experiment using exercise-trained rats (0.002 g/kg body weight and 0.006 g/kg body weight) was lower than that of the present experiment using high-fat diet mice (0.05 g/kg body weight). However, the net effect of OFE needs to be elucidated in order to validate a possible mechanism regarding the synergistic action of GPE plus OFE in diet-induced obese mice, which is in progress at our laboratory presently.

Along with its active role in regulating energy balance, WAT secretes a variety of adipocytokines that have important physiologic functions, including cytokines (TNF-*α* and IL-6), chemokines (MCP-1), leptin, PAI-1, resistin, adiponectin and adipsin [28]. Leptin is principally produced by adipocytes and PAI-1 is also largely produced by visceral adipocytes [29]. In addition, several proinflammatory cytokines and chemokines such as IL-6, TNF-*α* and MCP-1 are secreted by adipocytes (as well as nonadipocyte cells such as macrophages), thus contributing to the chronic inflammatory state often observed in obesity [30]. The circulating levels of several adipocytokines, such as TNF-*α*, IL-6, MCP-1, leptin, and PAI-1, are elevated in obesity and are reduced with weight loss [31–33]. In particular, visceral WAT from obese subjects has been found to secrete higher levels of TNF-*α*, IL-6, MCP-1, leptin, and PAI-1 but lower levels of adiponectin compared to lean subjects [34–37]. We found that supplementation with GPE + OFE significantly lowered the plasma leptin, PAI-1, MCP-1, TNF-*α* and IL-6 levels compared to the control group although it did not alter the plasma adiponectin level. In addition, GPE plus OFE significantly downregulated mRNA expression of proinflammatory cytokines (TNF-*α*, IL-6) and chemokines (MCP-1) as well as its transcription factor NF-*κ*B in epididymal WAT. There was a strong positive correlation between circulating and adipose tissue levels of IL-6 and TNF-*α* in obese individuals, which suggested that circulating proinflammatory cytokines originate from adipose tissue [38]. MCP-1 mRNA expression was also increased in the epididymal WAT of diet-induced obese mice, along with the plasma MCP-1 level [39]. The transcription factor NF-*κ*B is well known to up-regulate the mRNA expression of many inflammation-related genes, including TNF-*α*, IL-6 and MCP-1 [40].

NAFLD is characterized by the accumulation of fat and inflammatory changes in the liver. Visceral adiposity is closely related to NAFLD [41] because free fatty acids and proinflammatory factors released from visceral WAT are directly transported to the liver by the portal vein and may contribute to hepatic steatosis and inflammation [42]. Higher concentrations of circulating free fatty acids are observed in obese persons and animals, particularly those with abdominal obesity [43]. Excess free fatty acid availability in plasma leads to increased hepatic free fatty acid uptake and syntheses of triglyceride, which can be stored as lipid droplets within hepatocytes. In subjects with NAFLD, nearly 60% of the hepatic triglycerides were derived from circulating free fatty acid in a fasting state, suggesting plasma free fatty acid is the main contributor to triglyceride accumulation in the liver [43]. Interestingly, in the present study, the supplementation of GPE + OFE significantly decreased the plasma free fatty acid level as well as hepatic free fatty acid and triglyceride contents in HFD-fed mice. The hepatic free fatty acid level was higher in subjects with morbid obesity or alcoholic liver disease compared to controls [44]. Larter et al. [45] suggested that hepatic free fatty acid accumulation itself is a lipotoxic insult for liver injury in fatty liver disease. We also found that the GPE + OFE diet significantly downregulated mRNA expression of lipogenic transcription factor PPAR*γ* and their target genes, CD36, FAS, ACC1, SCD1, ME and LPL, in the liver. The expression of genes involved in fatty acid oxidation (ACOX1 and UCP2) was also decreased in GPE + OFE group compared to the control group, which seemed to be meditated by adaptation process of energy metabolism. Similar to our results, genetic ablation of PPAR*γ* in *ob/ob* mice significantly downregulated expression of hepatic genes involved in fatty acid *β*-oxidation (PPAR*α*, ACOX, and UCP2) as well as fatty acid uptake (CD36) and syntheses (FAS, SCD1 and LPL) [46]. Furthermore, GPE + OFE feeding markedly decreased hepatic cholesterol accumulation, in part, by suppressing mRNA expression of hepatic HMGR and ACAT which are rate-limiting enzymes of cholesterol syntheses and esterification, respectively. Accordingly, a decrease in plasma free fatty acids and hepatic fatty acid uptake, *de novo* fatty acid and cholesterol syntheses may partially explain the improvement of hepatic lipid accumulation and the subsequent lipid droplet formation observed in GPE + OFE-supplemented obese mice. Although the mRNA expression of fatty acid oxidation-related genes such as ACOX1 and UCP2 in liver was downregulated in GPE + OFE-supplemented mice compared to that in the control mice, the hepatic fatty acid *β*-oxidation was activated by GPE alone and with OFE, indicating presence of the posttranslational regulation. One possibility is that GPE with or without OFE may promote fatty acid *β*-oxidation by regulating the ratio of NADH/NAD^+^ and acetyl CoA/CoA, since an increase in the NADH/NAD^+^ or acetyl CoA/CoA ratios results in inhibition of fatty acid *β*-oxidation [47]. To clearly understand whether GPE alone and GPE with OFE regulate the level of the products formed during *β*-oxidation, further experiments are needed to be performed. 

Referring to the previously ment coned, proinflammatory factors from portal circulation, potentially produced in visceral WAT, might affect hepatic steatosis and inflammation [42]. Visceral WAT was independently associated with hepatic inflammation and fibrosis in NAFLD subjects and serum IL-6 levels, which correlated with visceral fat, independently predicting an increase in hepatic inflammation [1]. Leptin is also reported to be a mediator of liver fibrosis after chronic liver injury in mouse models [48]. The liver can also produce and secrete inflammatory cytokines. Cai et al. [49] suggested that the presence of hepatic steatosis is closely related to chronic hepatic inflammation through NF-*κ*B activation and downstream cytokine production. Selective hepatocellular activation of NF-*κ*B increased production of hepatic inflammatory cytokines such as IL-6 and TNF-*α* in mice to a similar extent as observed in HFD-fed obese mice, whereas liver-specific NF-*κ*B inhibition prevented HFD-induced inflammatory gene expression [49]. IL-6 is the inflammatory cytokine that is synthesized in the liver as well as the WAT and increases in NAFLD [50]. TNF-*α* also plays a major role in the development of NAFLD by upregulating lipogenic gene expression, increasing mitochondrial generation of reactive oxygen species, promoting hepatocyte apoptosis and recruiting inflammatory cells to the liver [51, 52]. The inhibition of TNF-*α* improved NAFLD in HFD-fed *ob/ob* mice [53], while TNF-*α* receptor-deficient mice protected against hepatic steatosis [54]. In the present study, similar to WAT, a combination of GPE and OFE significantly downregulated the proinflammatory transcription factor NF-*κ*B and its target genes, TNF-*α* and IL-6, in the liver which may be one potential mechanism for improving NAFLD in HFD-induced obese mice. These protective effects against NAFLD were also supported by significant decreases in plasma levels of ALT and AST in GPE + OFE-supplemented obese mice, since the elevated liver aminotransferase (ALT and AST) is positively correlated to 90% patients with nonalcoholic steatohepatitis, characterized by a liver lipid accumulation combined with hepatic inflammation [55]. 

## 5. Conclusions

**Figure 3:**
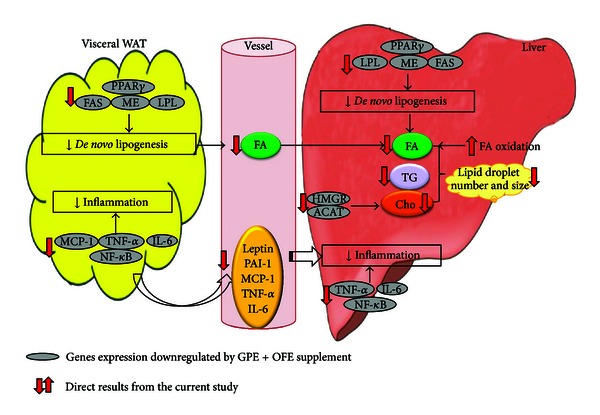
Schematic of the proposed mechanism underlying the protective effect of GPE combined with OFE on lipid metabolism and inflammation in the liver and WAT adipose tissue of HFD-fed mice. Supplementation with GPE + OFE significantly lowered body weight gain and body fat mass by partly suppressing mRNA expression of lipogenic genes (FAS, ME, and LPL) and its transcription factor (PPAR*γ*) in epididymal WAT. The mRNA expression of hepatic genes involved in fatty acid and cholesterol syntheses (PPAR*γ* FAS, ME, LPL, HMGR, and ACAT) was also downregulated by supplementation with GPE + OFE, whereas GPE + OFE supplementation activated hepatic fatty acid oxidation, leading to decreased hepatic lipid accumulation. Furthermore, the supplementation of GPE + OFE significantly decreased the levels of plasma adipocytokines (leptin, PAI-1, MCP-1, TNF-*α*, and IL-6) as well as the mRNA expression of proinflammatory transcription factor, NF-*κ*B, and its target genes, including MCP-1, TNF-*α* and IL-6, in the liver and epididymal WAT, which may be related to the improvement in obesity and NAFLD. ACAT: acyl-CoA:cholesterol acyltransferase; Cho: cholesterol; FA: fatty acid; FAS: fatty acid synthase; HMGR: 3-Hydroxy-3-Methylglutaryl-CoA Reductase; LPL: lipoprotein lipase; IL-6: interleukin-6; MCP-1: monocyte chemotactic protein-1; ME: malic enzyme; NF-*κ*B: nuclear factor-*κ*B; PPAR*γ*: peroxisome proliferator-activated receptor *γ*; TG: triglyceride; TNF-*α*: tumor necrosis factor-*α*.

The present study demonstrated that combined supplementation with GPE and OFE significantly ameliorated adiposity and hepatic steatosis more than the responses to GPE alone. It seemed that the expression of genes involved in the multiple steps of lipid accumulation and inflammation in the liver and WAT was downregulated in response to the GPE plus OFE diet (Figure 3). These findings provide information on the molecular mechanisms by which the combination of GPE and OFE influences the regulation of body fat and hepatic fat accumulation and inflammation, and further suggest that GPE plus OFE may have a potential use for regulating adiposity and NAFLD in obese mice.

## Figures and Tables

**Table 1:** Composition of experimental diets (unit: % of diet).

Ingredients	CON	GPE	GPE + OFE
Casein	20	20	20
D, L-Methionine	0.3	0.3	0.3
Sucrose	36.996	36.496	36.45
Cellulose	5	5	5
AIN mineral^1^	4.2	4.2	4.2
AIN vitamin^2^	1.2	1.2	1.2
Choline bitartrate	0.2	0.2	0.2
Corn Starch	11.1	11.1	11.1
Lard	17	17	17
Corn oil	3	3	3
Cholesterol	1	1	1
tert-butylhydroquinone	0.004	0.004	0.004
Grape extract		0.5	0.5
Omija extract			0.05

Total	100	100	100

CON: high-fat diet control; GPE: high-fat diet plus grape pomace extract (0.5%, w/w); GPE + OFE: high-fat diet plus grape pomace extract (0.5%, w/w) combined with omija fruit extract (0.05%, w/w). ^1^AIN-76 mineral mixture (grams/kg): calcium phosphate 500, sodium chloride 74, potassium citrate 2220, potassium sulfate 52, magnesium oxide 24, manganous carbonate 3.5, ferric citrate 6, zinc carbonate 1.6, cupric carbonate 0.3, potassium iodate 0.01, sodium selenite 0.01, chromium potassium sulfate 0.55, sucrose 118.03, ^2^AIN-76 vitamin mixture (grams/kg): thiamin HCl 0.6, riboflavin 0.6, pyridoxine HCl 0.7, niacin 3, calcium pantothenate 1.6, folic acid 0.2, biotin 0.02, vitamin B_12_ 1, vitamin A (500,000 U/gm) 0.8, vitamin D_3_ (400,000 U/gm) 0.25, vitamin E acetate (500 U/gm) 10, menadione sodium bisulfite 0.08, sucrose 981.15.

**Table 2:** Effects of GPE alone or combined with OFE on food intake, body weight, plasma adipokines and lipids levels in HFD-fed mice.

	CON	GPE	GPE + OFE
Food intake (g/day)	3.23 ± 0.08	3.00 ± 0.10	3.21 ± 0.05
Initial body weight (g)	18.95 ± 0.07	18.65 ± 0.45	18.85 ± 0.51
Final body weight (g)	33.74 ± 1.03	31.87 ± 0.81	31.15 ± 0.94
Adiponectin (*µ*g/mL)	9.67 ± 0.27	10.43 ± 0.27	10.41 ± 0.31
Adipsin (*µ*g/m)	0.99 ± 0.04	1.00 ± 0.03	1.08 ± 0.03
Free fatty acid (mmol/L)	0.47 ± 0.04^a^	0.45 ± 0.04^a^	0.29 ± 0.04^b^
Triglyceride (mmol/L)	0.92 ± 0.09	1.03 ± 0.08	0.90 ± 0.03
Phospholipid (mmol/L)	2.24 ± 0.06	2.39 ± 0.08	2.27 ± 0.04
Total cholesterol (mmol/L)	4.21 ± 0.16	4.66 ± 0.28	4.16 ± 0.18
HDL cholesterol (mmol/L)	0.76 ± 0.06^a^	0.96 ± 0.09^b^	0.84 ± 0.03^ab^
HTR (%)	17.99 ± 1.00	20.30 ± 0.89	20.31 ± 0.59
AI	4.69 ± 0.34^a^	4.00 ± 0.21^b^	3.96 ± 0.15^b^

Data are mean ± SE (*n* = 10). ^ab^Means not sharing a common letter are significantly different among groups at  *P* < 0.05. CON: high-fat diet control; GPE: high-fat diet plus grape pomace extract (0.5%, w/w); GPE + OFE: high fat diet plus grape pomace extract (0.5%, w/w) combined with omija fruit extract (0.05%, w/w); HTR: HDL cholesterol/total cholesterol; AI atherogenic index; (total-cholesterol − HDL cholesterol)/HDL cholesterol.
